# DTS-Net: Depth-to-Space Networks for Fast and Accurate Semantic Object Segmentation

**DOI:** 10.3390/s22010337

**Published:** 2022-01-03

**Authors:** Hatem Ibrahem, Ahmed Salem, Hyun-Soo Kang

**Affiliations:** 1 Department of Information and Communication Engineering, School of Electrical and Computer Engineering, Chungbuk National University, Cheongju-si 28644, Korea; hatem@cbnu.ac.kr (H.I.); ahmeddiefy@cbnu.ac.kr (A.S.); 2 Electrical Engineering Department, Faculty of Engineering, Assiut University, Assiut 71515, Egypt

**Keywords:** convolutional neural networks, semantic segmentation, real-time computer vision

## Abstract

We propose Depth-to-Space Net (DTS-Net), an effective technique for semantic segmentation using the efficient sub-pixel convolutional neural network. This technique is inspired by depth-to-space (DTS) image reconstruction, which was originally used for image and video super-resolution tasks, combined with a mask enhancement filtration technique based on multi-label classification, namely, Nearest Label Filtration. In the proposed technique, we employ depth-wise separable convolution-based architectures. We propose both a deep network, that is, DTS-Net, and a lightweight network, DTS-Net-Lite, for real-time semantic segmentation; these networks employ Xception and MobileNetV2 architectures as the feature extractors, respectively. In addition, we explore the joint semantic segmentation and depth estimation task and demonstrate that the proposed technique can efficiently perform both tasks simultaneously, outperforming state-of-art (SOTA) methods. We train and evaluate the performance of the proposed method on the PASCAL VOC2012, NYUV2, and CITYSCAPES benchmarks. Hence, we obtain high mean intersection over union (mIOU) and mean pixel accuracy (Pix.acc.) values using simple and lightweight convolutional neural network architectures of the developed networks. Notably, the proposed method outperforms SOTA methods that depend on encoder–decoder architectures, although our implementation and computations are far simpler.

## 1. Introduction

Semantic segmentation is an important task in computer vision, as it constitutes pixel-wise classification of an image to mask each object in the scene. Hundreds of applications, such as autonomous driving, robotics, medical diagnostics, image editing, and augmented reality applications, incorporate semantic segmentation. Recent studies on semantic segmentation have achieved promising results using convolutional neural networks (CNNs), particularly encoder–decoder CNN architectures. In such architectures, the semantic segmentation task is modeled in two stages: the encoding stage, in which the image is down-sampled to obtain its deep semantic features, and the decoding stage, in which the semantic features are up-sampled to obtain a semantic segmentation mask of the same size as the input image. Encoder–decoder architecture can achieve highly accurate segmentation results; however, the decoder stage adds considerable computational complexity to the overall model and the input image size of such models is usually small. Furthermore, the decoding-stage reconstruction is inefficient because de-convolution or up-sampling layers are used, which eliminate small details and sometimes propagate noise.

The semantic segmentation problem can be seen as an image-to-image translation problem, as the aim of this task is to construct segmentation masks equivalent to each object in the input image. Further, the objects in the segmentation mask have the same boundaries as the input image; thus, the segmentation mask can be considered as an alternative to the image and, hence, the segmentation task can be performed using image reconstruction techniques. The efficient sub-pixel CNN [1] has shown promising results in image and video super-resolution tasks because of its depth-to-space (DTS) layer, from which a high-resolution image can be reconstructed from many low-resolution images. This layer performs image reconstruction through pixel reordering of the low-resolution feature maps obtained from the CNN network, in order to form super-pixels of the high-resolution image with very accurate borders and object details. Thus, the DTS layer efficiently obtains accurate semantic segmentation masks with clear borders, at far higher speeds than the traditional encoder–decoder architectures usually used for segmentation tasks. The DTS layer has many advantages, including its lower computation count and considerably higher accuracy compared with the decoder stage of the encoder–decoder architecture. Therefore, modeling can be accelerated, while high segmentation accuracy is maintained.

Vision transformers, which apply a transformer network to images in a manner similar to natural language processing, are a recent achievement in computer vision. Vision transformers allow parallelization in the processing of a sequence dataset or a sequence of patches of the target image. This is achieved through positional encoding, which allows the network to learn the position of the patch in the input “big image”. Vision transformer-based methods achieve better accuracy than CNN-based methods for classification, segmentation, and object detection tasks without increased computational cost.

In this study, we propose the DTS-Net deep network for semantic segmentation using a sub-pixel CNN, to address the semantic segmentation complexity problem that arises for encoder–decoder architectures with or without attention methods while retaining high segmentation accuracy. We also present DTS-Net-Lite, a lightweight version of this network. Our contributions can be summarized as follows:

- Rather than a pixel-wise classification problem, we treat the semantic segmentation problem as an image-to-image translation problem through regression using the DTS layer, and construct segmentation maps using the higher-resolution image reconstruction approach of the super-resolution task;
- We propose DTS-Net, a deep model that uses Xception architecture as a feature extractor for high-accuracy critical applications, as well as a small lightweight model, DTS-Net-Lite, for high-speed critical applications that uses MobileNetV2 architecture as a feature extractor;
- We reduce the typical decoding stage complexity for segmentation mask construction to that of the DTS image construction layer and show that this layer can construct segmentation masks with far lower computational cost and much higher precision than conventional CNN-based decoding architectures.
- We propose a new segmentation improvement technique namely nearest label filtration (NLF) to improve the segmentation by correcting the wrong predicted pixels by DTS-layer in the segmentation mask.

**Figure 1:**
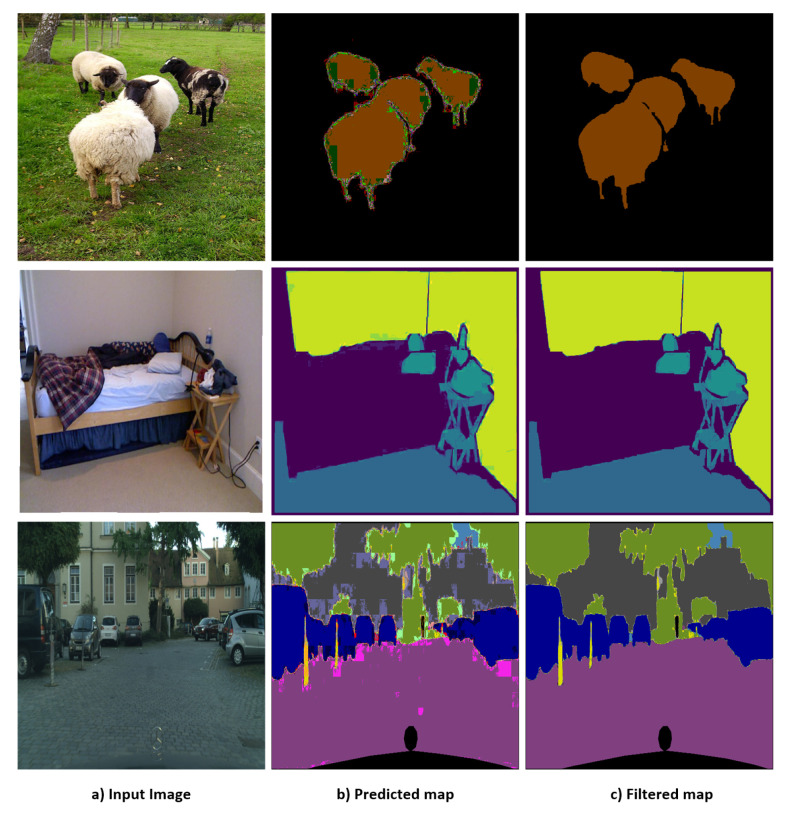
Sample predicted and filtered semantic segmentation results of proposed method: (top to bottom) samples from PASCAL VOC2012 [2], NYUV2 [3], and CITYSCAPES [4] benchmarks, respectively.

The proposed method achieves higher accuracy and speed than the recent semantic segmentation methods. Further, learning of highly detailed feature maps is possible as we depend on CNN architecture in addition to the DTS layer; this allows the construction of higher-resolution prediction maps. In addition, we explore the joint semantic segmentation and depth estimation task and achieve promising results. A preview of our results is shown in Figure 1. The remainder of this paper is structured as follows. Section 2 summarizes related work and Section 3 presents the proposed method and architectures. Section 4 and Section 5 discuss the training and test datasets and present the results with comparison to state-of-the-art (SOTA) methods, respectively. Section 6 discusses future work and Section 7 presents conclusions.

## 2. Related Work

Recent semantic segmentation studies have shown that encoder–decoder architecture can efficiently perform segmentation tasks. The first encoder–decoder architecture was the fully convolutional network (FCN) [5], in which the same architecture was used for image classification but the final dense layers were replaced with 1 × 1 convolutional layers having the same weights. The decoder stage was a simple up-sampling layer. The FCN obtained relatively good results, motivating further research on encoder–decoder architectures. Later, SegNet [6] was proposed as a deep encoder–decoder architecture. This architecture features pooling indices shared between the max-pooling layers in the encoder stage and the corresponding max-unpooling layers in the decoder stage. SegNet exhibited impressive semantic segmentation results on outdoor and indoor segmentation datasets. U-Net [7] is another impressive encoder–decoder architecture, which was proposed for microscopy cell segmentation in medical images. U-net suggests residual connections between corresponding layers in the encoder and decoder stages and, hence, achieves considerable segmentation accuracy.

The four versions of DeepLabVx also constitute considerable contributions to the semantic segmentation task. DeepLabV1 [8] tackled the problem of inefficient down-sampling through a wide field of view convolution using the Atrous convolution [8], which increases the spatial field of the convolutional window using the same weights as the normal convolution. In that work, the conditional random field (CRF) was also proposed, which uses an energy function derived from the summation of a unary potential term calculated from the probability distribution of the output label of each pixel, along with a binary potential term calculated from the correlation between pixel labels. In general, the CRF allows the model to learn small image details. With DeepLabV2 [9], Atrous Spatial Pyramid Pooling (ASPP) was proposed to enhance the model learning at multiple scales of the feature maps. In addition, the VGG16 [10] used in DeeplabV1 was replaced with ResNet [11], which yielded better performance. The ASPP was further improved for DeepLabV3 [12], with the use of different sampling rates in the ASPP in a cascaded manner. Finally, for DeepLabV3+ [13], the encoder architecture was replaced with a depth-wise separable convolution-based architecture, and Aligned Xception was adopted. The latter is a modified version of Xception [14] that replaces the max-pooling layers in the original architecture with strided convolutional layers with more Xception blocks; this facilitates higher accuracy and speed during processing.

Zhao et al. proposed the pyramid scene parsing network (PSPNet) [15], which adopts a pyramid parsing module that learns the global context of the image through region-based aggregation. Pyramid pooling is employed to learn the context of the image from the final small feature maps. Later, Zhao et al. presented the image cascade network (ICNet) [16], which performs high-speed semantic segmentation on high-resolution images using cascade feature fusion. The latter approach mixes the features obtained from the input image on different scales in a method called cascade label guidance. Up-sampling layers are then used to resize the image to the input image size. As another approach, the bilateral segmentation network (BiSeNet) [17] performs real-time semantic segmentation using an architecture with two paths. The first is a spatial path for spatial information preservation and the other is a context path for general context learning through down-sampling. The features obtained by the two paths are then combined via a feature fusion technique. ResNeSt, developed by Zhang et al. [18], applies channel-wise attention to different network branches to learn diverse feature representations and cross-feature information. Shi et al. [19] proposed Hierarchical Parsing Net for semantic segmentation which enhances scene parsing by learning the global scene information and the contextual relation between objects in the scene using a deep neural network. Chen et al. [20] proposed a one-shot semantic segmentation method which uses the multiclass labels information during training to encourage the network to learn more accurate semantic features of each category and they also proposed the pyramid feature fusion module to mine the fused features of the objects and a self-prototype guidance branch to support the segmentation task. Although all the previously mentioned methods presented challenging results, they adopted inefficient decoding stages that eliminate the object details and introduce some noise.

Zoph et al. [21] proposed pre-training and self-training techniques using stronger augmentation on ImageNet [22] across the different image sizes using EfficientNet [23] architecture, and showed that pre-training and self-training are mutually beneficial and improve the accuracies of both the semantic segmentation and object detection tasks. Further, Rashwan et al. [24] proposed dilated SpineNet or SpineNet-Seg, which is a neural architecture search discovered network from DeepLabv3. In this approach, the scale permuted networks originally used for object detection in the semantic segmentation task are evaluated, adopting a customized dilation ratio per block. Bai et al. [25] proposed the multiscale deep equilibrium model (MDEQ), MDEQ backpropagates through the equilibrium points of multiple scale features simultaneously using simple differentiation to avoid storing intermediate states, they attained a high segmentation accuracy on CITYSCAPES however, their model has high computational complexity. Termritthikun et al. [26] proposed EEEA-Net in which they employed a neural architecture search method to search an optimized model with the lowest number of parameters by using an early exit population initialization algorithm. They could achieve an average accuracy segmentation model but the number of the parameters of their model was extremely low. Ding et al. [27] recently proposed RepVGG in which they adopted a VGG-like architecture composed of a stack of 3×3 convolution and Relu. RepVGG models ran much faster than ResNet-50 or ResNet-101 with higher accuracy on the classification and semantic segmentation tasks. It is important to note that Aich et al. [28] initiated the direction of using depth-to-space for segmentation when they employed it to perform binary segmentation for satellite maps in DeepGlobe dtaset [29] using ResNet and VGG16 backbones but their implementation was not efficient enough and our model can produce better quality segmentation than their model.

Other methods [30,31] employed depth information to support the semantic segmentation task. Kang et al. [30] proposed a depth adaptive deep neural network for semantic segmentation using a depth-adaptive multiscale convolutional layer consisting of the adaptive perception neuron and the in-layer multiscale neuron to adjust the receptive field at each spatial location and to apply the different size of the receptive field at each feature space to learn features at multiple scales, respectively. Gu et al. [31] proposed Hard Pixel Mining for semantic segmentation using a multiscale loss weight map generated by the depth data to enforce the model to pay more attention to the hard pixels in segmentation. They employed the depth data during the training step only and did not use it in the testing step. Other studies have shown the ability of CNN models to perform the joint task of semantic segmentation and depth estimation. For example, Mousavian et al. [32] proposed a multi-scale fully convolutional CNN for simultaneous semantic segmentation and monocular depth estimation. In this architecture, a CNN model is coupled with a fully connected conditional random field (CRF) to obtain the contextual relation and the interactions between the semantics of the image and depth cues. Zhang et al. [33] developed joint task-recursive learning (TRL) for semantic segmentation and depth estimation and showed that TRL can recursively refine the results from both tasks using a task attention module. Further, a hybrid CNN for depth estimation and semantic segmentation (HybridNet) was developed by Lin et al. [34]; this network functions by sharing the parameters that can yield mutual improvement for each task. Finally, He et al. [35] proposed semantic object segmentation and depth estimation (SOSD)-Net for application to monocular images; in this approach, semantic objectness is used to perform image processing based on the geometric relationship between the two tasks. The aligned Xception architecture is employed.

In all of the related work, the studies adopted mainly encoder-decoder approaches with some methods adopted extra attention methods or transformers. The proposed method mainly eliminates the need for a complex decoding stage which is computationally expensive, eliminates some of the image details, and propagates noise. Hence, we propose a simple and fast approach for dense prediction. We use the DTS-layer as the decoding stage to directly construct a dense map out of small feature maps extracted from an encoding stage.

## 3. Proposed Method

**Figure 2:**
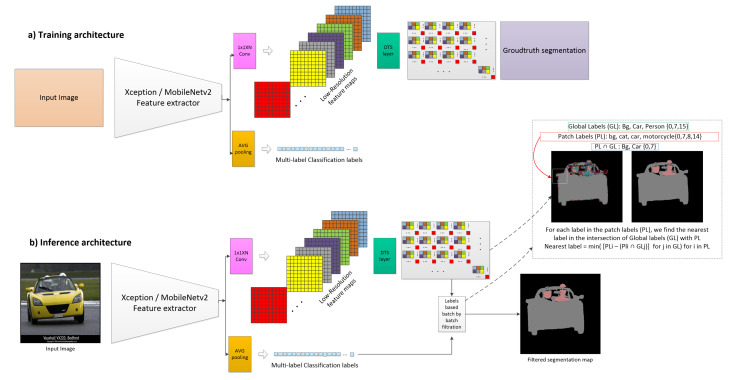
The architecture of the proposed method. With an Xception (DTS-NET) or MobileNetV2 (DTS-Net-Lite) backbone, the DTS layer (module) is added after a $1\times 1\times r^{2}$ convolutional layer. The pixels in the $h\times w\times r^{2}$ low-resolution feature maps are arranged in an rh×rw segmentation map. Another branch features global average pooling and a fully connected layer is added for multi-label classification. (**a**) Training architecture of the proposed method and (**b**) inference architecture, which features one additional step, that is, the nearest label filtration (NLF). NLF is a patch-by-patch image filtration based on the global labels of the image and the patch labels. The noisy pixels with labels that are not included in the global image labels are mapped to the nearest neighbor in the intersection between the patch and global labels using a simple iterative difference operation.

The proposed method, which is illustrated in Figure 2, consists of three main components:

- The feature extractor CNNs: These CNNs extract the image semantics and deliver deep feature maps. Both proposed architectures, DTS-Net and DTS-Net-Lite, are presented in this section. We also discuss our reasons for choosing depth-wise separable convolution-based architectures.
- DTS layer: This layer aggregates the small feature maps to form a higher-resolution segmentation map (or depth map).
- The nearest label filtration (NLF): This filtration employs multi-label classification labels to filter the segmentation mask patch by patch.

### 3.1. Feature Extractor Architectures

The feature extractor is the most important part of a dense prediction CNN model, as the dense predictions are highly dependent on the extracted features. Depth-wise separable convolution was first presented by Chollet [14] and was clearly shown to be much faster and more efficient than normal convolution. Depth-wise separable convolution-based architectures (such as Xception and MobileNetV2 [36]) can attain high accuracy for ImageNet classification and other tasks (such as object detection and semantic segmentation) with a significantly low parameter number and low floating-point operations count compared with conventional CNN-based architectures.

**Xception** [14] was also proposed by Chollet. This unique convolution approach consists of two main modules: depth-wise and point-wise convolution. In depth-wise convolution, the convolution is performed for each channel separately, whereas point-wise convolution involves a 1 × 1 convolution through the channels; the latter can be regarded as channel projection or dot-product multiplication between channels. As regards the computational cost of depth-wise separable convolution [36], it is clearly apparent that this convolution has $\frac{1}{N}+\frac{1}{D_{k}^{2}}$ fewer parameters than conventional convolution, where *N* is the number of channels and $D_{k}$ is the kernel size.

**Figure 3:**
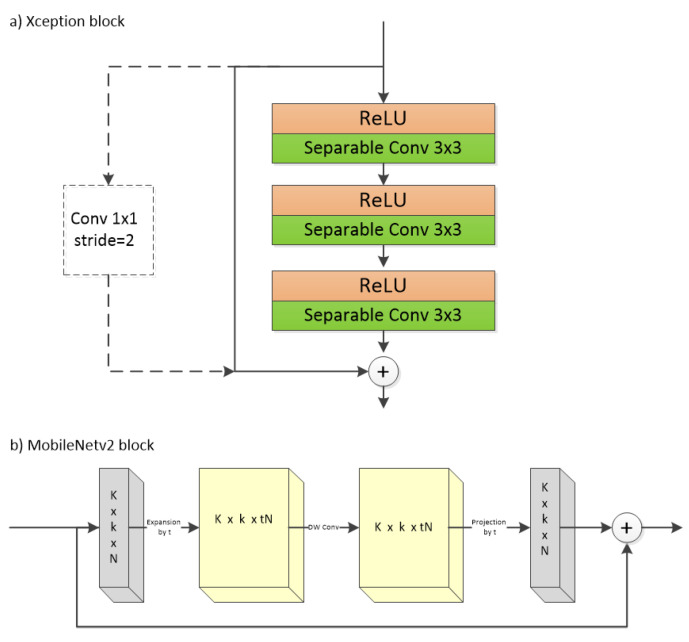
(**a**) Xception block. There are three depth-wise separable convolutions, each having prior ReLU activation. A residual connection connects the block start and end in the entry-flow stage. A 1 × 1 convolution is implemented in the middle-flow stage. (**b**) MobileNetV2 block. An expansion module is followed by a Depth-wise separable convolution layer, then a projection module was added with a residual connection between the beginning and the end of the block.

The original Xception architecture proposed for Imagenet classification consists of three stages. The main building block of the model is the Xception block, which involves two or three depth-wise separable convolutions with a kernel size of 3 × 3 and rectified linear unit (ReLU) activation with or without max-pooling at the end. Residual connections are used to connect the block from start to end. The first stage involves the entry flow, which has three Xception blocks with max-pooling at the end of each block. The second stage involves the middle flow, which has eight Xception blocks without max-pooling. Finally, the third stage pertains to the exit flow, which has one Xception block with max-pooling and then two depth-wise separable convolutions. The Xception block details are shown in Figure 3a. The final feature map depth is 2048. In our proposed approach, we then add a 1 × 1 × 1024 (i.e., 32 × 32) convolution to reduce the depth from 2048 to 1024, so as to aggregate a segmentation map that is 32 times the width and 32 times the height of the final feature maps after the DTS layer. As a second branch, we add global average pooling and then a fully connected layer with sigmoid activation to obtain the multi-label classification predictions. The Xception feature extractor is incorporated in the main network proposed in this work, that is, DTS-Net.

**MobileNetV2** [36] is the other architecture employed in this study and is implemented in DTS-Net-Lite to accelerate our approach for real-time application on low-computational-power devices such as embedded devices or mobile phones. Like Xception, MobileNetV2 adopts depth-wise separable convolution as the main building module, to benefit from the cheap computational cost of this module along with other features that further decrease the computation. In MobileNetV2, inverted residuals and linear bottlenecks are implemented. The feature maps can be encoded in a low-dimensional subspace using the bottlenecks with a linear operation. A bottleneck consists of an expansion module (1 × 1 convolution with a higher number of filters with expansion factor *t*), followed by depth-wise separable convolution with a 3 × 3 kernel and, finally, a projection module (1 × 1 convolution with a lower number of filters with the same factor *t*). In [36], t=6 was employed as the expansion factor. Each of the three modules features patch normalization and ReLU6 activation (i.e., ReLU with the maximum value clipped to 6). The inverted residuals form a skip-connection between the bottleneck start and end, which have the same channel depth. This gives the network access to earlier features before entering the bottleneck block. MobileNetV2 originally consisted of seven sequential MobileNet blocks (a MobileNetV2 block is shown in detail in Figure 3b), followed by a 1 × 1 × 1280 convolution. In our proposed approach, we add a 1 × 1 × 1024 convolution to reduce the depth to 1024 (32 × 32) in order to construct a dense map using the DTS layer. We also add a global average pooling layer after the 1 × 1 × 1280 convolution, which is followed by a fully-connected layer with sigmoid activation for multi-label classification. As noted above, we employ MobileNetV2 as the feature extractor of the lightweight version of the proposed method, that is, DTS-Net-Lite.

We believe that our choice of depth-wise separable convolution-based architecture is key to the high accuracy and speed achieved in this work. The depth-wise convolution (DW-Conv) is perfectly compatible with depth-to-space image construction as DW-Conv extracts information from each channel separately, allowing small details in each channel (depth-axis) to be captured. Then, the DTS layer reconstructs the image from the aggregation of the information in each channel, as discussed in detail in the next section. This explains the high accuracy of the depth-wise separable convolution-based architecture combined with the DTS dense map reconstruction.

### 3.2. Efficient Sub-Pixel Convolutional Neural Networks

Efficient sub-pixel CNN [1] was originally proposed for image and video super-resolution, in which high-resolution images are constructed from lower-resolution images through DTS aggregation. In this approach, pixels with the same location in the low-resolution image channels are mapped and re-ordered to form a super-pixel in the high-resolution image. DTS aggregates small feature maps of dimension *h* × *w* × $r^{2}$ to form a final high-resolution dense map of size rh × rw (as a segmentation or depth map), where *h*, *w*, and $r^{2}$ are the feature-map width, height, and depth, respectively. The feature maps extracted from the feature extractor CNN are passed to the 1 × 1 × $r^{2}$ convolutional layer, which gives a total activation patterns of $r^{2}$ with size $\frac{W}{r}$ and $\frac{H}{r}$, where *W* and *H* are the width and height of the input image, respectively. The final reconstructed dense map is also produced. The DTS module then rearranges the elements of the tensor $\frac{W}{r}$ × $\frac{H}{r}$ × $r^{2}$ to a tensor of shape $r.\frac{W}{r}$ × $r.\frac{H}{r}$, which is equivalent to the input image size of *W* × *H*. This operation is illustrated visually in Figure 2. In this method, fast one-step up-sampling is performed, which can be mathematically expressed as

(1) $$MAP_{x,y}=F_{\left[x/r],[y/r],r.mod(y,r)+mod(x,r\right)},$$

where $MAP_{x,y}$ is the constructed dense map, *F* represents the feature maps extracted from the CNN and passed to the 1 × 1 × $r^{2}$ convolutional layer, *r* is the feature map depth, and operation mod is the modulus, which maps the pixels from the low-resolution feature maps to the super-pixel in the final dense map when mod(x,r)=0 or mod(y,r)=0.

In our implementation, we use r=32, as the Xception or MobileNetV2 feature extractors down-sample the features using the convolution and max-pooling to 1/32 of the input image size. Therefore, to reconstruct the final dense map at the same size as the input image, we use the 1 × 1 × 1024 convolutional layer, where $r^{2}=1024$, to obtain a final feature map count equal to $r^{2}$. This reconstruction layer can be trained simply with a regression loss (we use the mean absolute error), while inference (a rounding operation) is used to round the predicted pixels in the dense layer to the nearest integer in the case of semantic segmentation.

### 3.3. Nearest Label Filtration

As we use an image reconstruction technique, the reconstructed segmentation map introduces a reconstruction noise, particularly at the borders of the objects in the image. Hence, mislabeled pixels are introduced. In detail, these noisy pixels are usually mislabeled with a label neighboring the true label. During the training stage, the network attempts to learn the true label as accurately as possible; however, an abrupt color change occurs at a border, causing mislearning of the border pixels. This problem is particularly notable when the CNN down-samples the image using max-pooling, and some details disappear from the final feature maps. To solve this problem, we propose Nearest Label Filteration (NLF), which aims to correct those noisy pixels by mapping them to the nearest classification label using a patch-by-patch technique. We train the network for multi-label classification besides segmentation; thus, we use the classification labels of the image (global labels) to filter the noisy pixels in the segmentation map. We divide the image into patches (imperially, 10 × 10 and 8 × 8 are the best patch sizes). For each patch, we obtain the unique labels that exist in the patch (patch labels). Then, we find the intersection between the patch labels (PL ) and global labels (GL ) of the image to obtain the true labels (TL ):

(2) TL=GL∩PL

By measuring the distance between each PL and the TL, we can find the distance array for all PL as follows:

(3) $$dist\_arr=|PL_{i}-TL_{j}|$$

for *i* in $\Omega_{PL}$ space and *j* in $\Omega_{GL}$ space. Then we can estimate the nearest label in TL corresponding to the minimum distance in dist_arr and overlay the noisy pixel with the nearest label (NL ), as follows:

(4) $$NL_{i}=TL\left[argmin\left(dist\_arr\right)\right]$$

This filtration method efficiently removes the noise from the image and provides clear edges. However, performance degradation occurs in extremely dense cases where the segmentation mask has a large number of classes and almost all pixels have widely differing labels (e.g., for the Cityscapes [4] dataset, in which the scenes are very crowded with small labels and many different classes). However, the method performs very well on the Pascal VOC2012 and NYUV2 datasets, in general. Figure 1 presents sample images that clearly show the difference before and after filtration. Figure 2 presents a visual illustration of the filtration step as a component of the overall proposed method.

### 3.4. Loss Function

The loss function for the proposed CNNs is divided into two separate loss functions: one for learning the semantic segmentation mask reconstruction and the other for multi-label classification. The loss for semantic segmentation reconstruction is the mean absolute error (MAE), which is expressed as follows:

(5) $$SegLoss=\frac{1}{r^{2}hw}\sum_{x=1}^{rw}\sum_{y=1}^{rh}\left|S_{x,y}^{GT}-S_{x,y}^{Const}\right|,$$

where $S_{x,y}^{GT}$ is the ground-truth pixel in the semantic segmentation mask and $S_{x,y}^{Const}$ is the pixel in the constructed semantic segmentation mask. The other loss is the multi-label binary cross-entropy (BCE) loss for *N*-classes of the dataset. This classification loss can be defined as

(6) $$ClassLoss=\sum_{i=1}^{N}\left[-p_{i}log\left(q_{i}\right)+\left(1-p_{i}\right)log\left(1-q_{i}\right)\right],$$

where *p* is the ground-truth label probability (0 or 1) and *q* is the predicted label probability. The multi-label binary cross-entropy loss is used to predict the objectness of each class independently with sigmoid activation which outputs a value in the range of 0 to 1 equivalent to the probability of the existence of each object. The employment of MAE for segmentation map regression and the multi-label classification for mask filtration improves the speed, it lowers the number of computations needed for obtaining the segmentation map as it constructs the segmentation map directly like an image with a single channel instead of predicting N channels image (where N is the number of classes) which is computationally expensive since the final segmentation maps have the same size of the input image which is relatively a high-resolution image (1024×512 in case of cityscapes). Additionally, mask filtration using multi-label classification (NLF) is much cheaper than constructing the N segmentation masks.

## 4. Experiments

In this section, we report the experiments performed to test the performance of the proposed method. These experiments include a segmentation quality comparison between different CNN backbones as feature extractors. We show the intermediate feature maps used to construct the segmentation maps. We also report semantic segmentation results for our proposed network DTS-Net and DTS-Net-Lite, and compare these results with those for state-of-the-art (SOTA) methods. Finally, we show that our method can be used for the joint task of semantic segmentation and depth estimation, as these two tasks are highly similar. The same training and testing environment and conditions are used for all experiments.

### 4.1. Semantic Segmentation Evaluation Metrics

To evaluate the proposed models on semantic segmentation, we use two evaluation metrics: mean intersection over union (mIOU ) and mean pixel accuracy (Pix.acc. ). The definition for those metrics is stated as below:

(7) $$mIOU=\frac{1}{N}\sum_{c=1}^{N}\frac{TP_{c}}{TP_{c}+FP_{c}+FN_{c}}$$

(8) $$Pix.acc.=\frac{1}{N}\sum_{c=1}^{N}\frac{TP_{c}+TN_{c}}{TP_{c}+TN_{c}+FP_{c}+FN_{c}},$$

where *N* is the number of class categories, TP (true-positive) represents the pixels that are truly predicted to be belonging to a class (*c* ), TN (true-negative) represents the pixels that are predicted to not belong to that class, FP (false-positive) the pixels which are falsely predicted to be belonging to a class, and FN (false-negative) the pixels which are falsely predicted to be not belonging to that class.

### 4.2. DTS-Net and DTS-Net-Lite

DTS-Net is the proposed deep architecture for semantic segmentation and employs Xception as the feature extractor CNN. In the experiment, we used relatively high-resolution images to obtain a segmentation mask with a fine border and high mean intersection over union. DTS-Net-Lite is the fast, lightweight version of the proposed method and employs MobileNetV2 as the feature extractor. In the experiment, we used approximately half the image size for DTS-Net to gain more processing speed; thus, some accuracy was sacrificed.

### 4.3. Benchmarks

To train and test our approach, we used three popular benchmarks for semantic segmentation, PASCAL VOC2012, NYUV2 Depth, and Cityscapes. Two of these benchmarks contain depth data.

**Figure 4:**
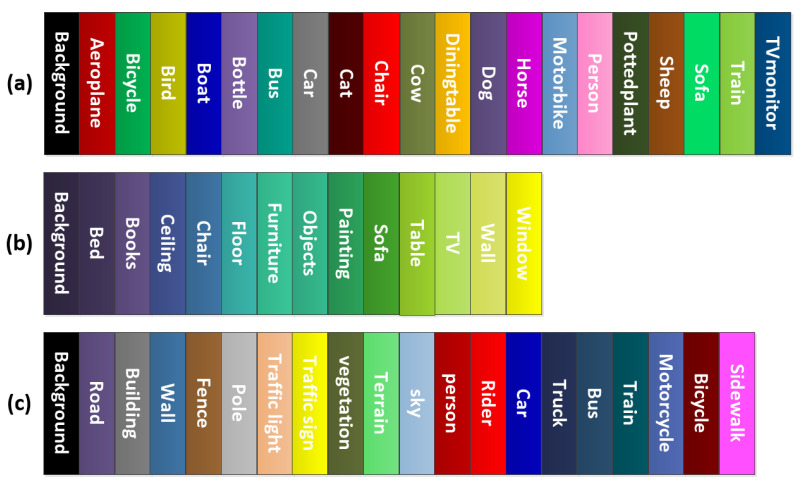
Class definitions and colormaps for the used benchmarks. (**a**) PASCAL VOC2012 [2] class definitions and colormap, (**b**) NYUV2 [3] class definitions and colormap, and (**c**) Cityscapes [4] class definitions and colormap.

**PASCAL VOC2012** [2] is a popular benchmark with 20 commonly seen classes. The dataset contains annotation for several computer vision tasks such as bounding-box object detection, semantic segmentation, instance segmentation, and action recognition. The segmentation data contain 2913 images of varying sizes: 1464 for training and 1449 for validation, the definition of the classes and the color used for each class’ mask is shown in Figure 4a. We resized the images to 480×480 and 256×256 for DTS-NET and DTS-Net-Lite training and testing, respectively. We trained the network as detailed in the previous section and constructed the segmentation map with the DTS layer at the same size as the image. This was achieved using feature map sizes of 15×15 and 8×8, both with a depth of 1024, for DTS-Net and DTS-Net-Lite, respectively (both architectures down-sampled the image to 1/32nd the input image size). In testing both networks, we used the same image size as in training.

**NYUV2 Depth** [3] is a benchmark for semantic segmentation and depth estimation. The image data are in RGB-D format and captured using a Kinect sensor. The dataset contains 1449 images of 13 indoor object classes, that is, 795 and 654 images for training and testing, respectively, with fixed image sizes, the definition of the classes, and the color used for each class’ mask is shown in Figure 4b. We trained and tested DTS-Net with the original image size (640×480) and used a 320×256 image size for DTS-Net-Lite. We trained both networks as described in Section 3, with feature map sizes before the DTS layer of 20×15 and 10×8, both with a depth of 1024, for DTS-Net and DTS-Net-Lite, respectively.

**Cityscapes** [4] is another benchmark for semantic segmentation and depth estimation, which contains urban street scenes in 50 cities, organized as 19 classes grouped into 8 categories. The dataset contains 5000 training images with fine segmentation masks, an additional 20,000 coarse annotated images, and 500 validation images, the definition of the classes and the color used for each class’ mask is shown in Figure 4c. We trained our models with the 5000 fine annotated images as we required high-resolution segmentation and depth estimation with fine borders. The original size of the images in the dataset was 2048×1024. We resized the training and testing images to half this size, that is, to 1024×512. This was still a high resolution for DTS-Net but allowed considerably faster training and inference. We then validated the model with the validation data. For DTS-Net-Lite, we resized the images to approximately one-third of the original size, that is, to 704×352, for higher-speed processing while sacrificing some accuracy. The feature map sizes before the DTS layer were 32×16 and 22×11 with a depth of 1024 for DTS-Net and DTS-Net-Lite, respectively.

### 4.4. Training and Testing Configuration

We trained the models using a desktop PC with an Intel Core i7-8700 CPU @3.20 GHz, Nvidia RTX3090 GPU, and 64 GB RAM. We used Tensorflow-Keras to implement the proposed method and all of the proposed models were initialized with the pre-trained version of the model on the ImageNet [22] dataset to accelerate the model fitting. We used Adam’s optimizer with an initial learning rate of 0.001, beta1 of 0.9, and beta2 of 0.999. We then evaluated the models using the same hardware configurations. All models were trained for approximately 1000 epochs.

### 4.5. Quality Comparison between Different CNN Backbones

**Figure 5:**
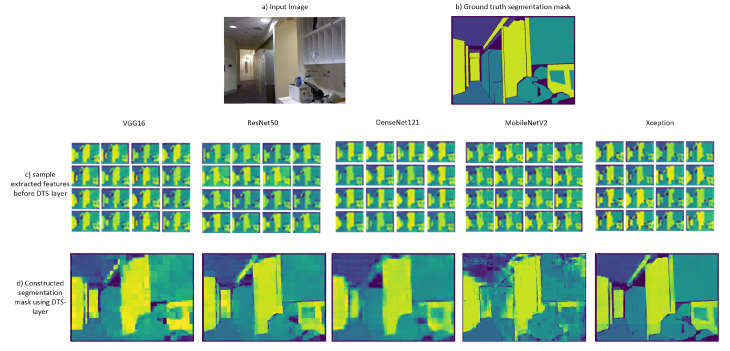
Quality comparison of (**a**) extracted feature maps before the DTS layer and (**b**) constructed segmentation mask using DTS layer (without NLF) between VGG16, ResNet50, DenseNet121, MobileNetV2, and Xception architectures.

**Figure 6:**
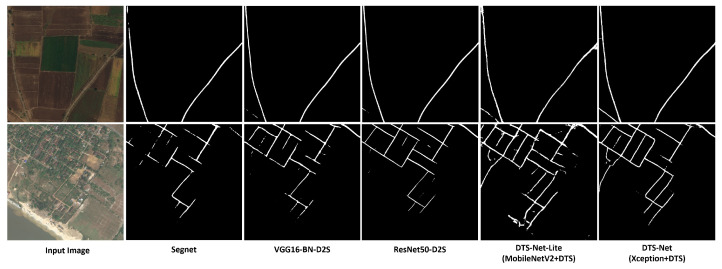
Quality comparison of the binary segmentation task on DeepGlobe [29] dataset between the models proposed by Aich et al. [28] (ResNet-D2S and VGG16-D2S) and SegNet [6] in the same paper and our models: DTS-Net and DTS-Net-Lite which uses Xception and MobileNetV2 as backbones, respectively.

We performed several experiments to test the ability of different CNN architectures to perform semantic segmentation using the proposed method. We trained three other architectures as feature extractors (VGG16 [10], ResNet50 [11], and DenseNet121 [37]) in addition to Xception and MobileNetV2. All were trained on the NYUV2 segmentation dataset with an image size of 640×480 for 1000 epochs. We then compared the features and the constructed segmentation masks obtained using the DTS layer (a sample test image is shown in Figure 5). For VGG16 and DenseNet121, the results showed features with inaccurate blurry edges, which were also apparent in the constructed segmentation masks. Better features and constructed masks with far more detail of the object edges and boundaries were extracted in the case of ResNet50 and MobileNetV2. The highest-detail features and the best-constructed segmentation maps were obtained for the Xception architecture. Therefore, we adopted this architecture as the feature extractor of our method. We adopted MobileNetv2 as the feature extractor for DTS-Net-Lite because that architecture exhibited average performance but the fewest multiplication/addition computations (MACs) (0.3 Giga; far lower than those of the other architectures), to achieve higher-speed processing. Table 1 compares the five architectures based on the mean pixel accuracy (Pix.acc. ) of each CNN with the DTS layer. This is a measure of the percentage of the correctly classified pixels in the predicted mask. The MAC and parameter numbers are also compared. To further support our selection and the proposed architecture, we trained DTS-Net on DeepGlobe satellite images dataset for road extraction using binary segmentation. We compared the obtained segmentation results from our architectures (using Xception and MobileNetV2 backbones) with ResNet-D2S and VGG16-D2S proposed in [28] (we also used Segnet results on DeepGlobe as the authors of this paper [28] trained SegNet [6] on DeepGlobe and added the results in their paper) in Figure 6. The comparison is simply a quality comparison of samples from the DeepGlobe validation satellite images as the validation server for this challenge is closed and there is no other way to validate the results. Our results on the binary segmentation of the satellite images show better quality in extracting more details than the results produced by ResNet-50 [11] and VGG16 [10] backbones in [28], even better than SegNet [6], which proves the excellent performance of the proposed architectures using Xception and MobileNetV2 backbones.

We selected depth-wise separable convolution as being suitable for the proposed DTS module based on the following main reason. The DTS layer constructs the final image using a pixel reordering technique through the channel axis to form super-pixels in the segmentation map, which highly depend on the feature-map quality. Depth-wise convolution extracts the features from each channel separately and then projects them using point-wise convolution. This approach extracts more accurate feature maps as the depth-wise convolution focuses on the data in each channel separately. These characteristics explain the good quality of the features extracted by the Xception and MobileNetV2 feature extractors.

### 4.6. Joint Semantic Segmentation and Depth Estimation

We further trained the proposed method on the simultaneous task of semantic segmentation and depth estimation. NYUV2 provides dense depth data captured by a Kinect sensor that is suitable for direct use, and CITYSCAPES provides disparity data that are equivalent to depth (as the disparity has a linear relationship with the depth). Here, we trained DTS-Net on the disparity without conversion. We trained our main model, DTS-Net, on NYUV2 with an image size of 640×480 and on Cityscape with an image size of 1024×512 for the depth estimation and semantic segmentation tasks. For the depth estimation, we added a third branch, which was identical to the branch for the segmentation branch, that is, a 1 × 1 × 1024 convolutional layer followed by a DTS layer to construct the depth map. We retained the two main branches, that is, the semantic segmentation branch and the multi-label classification branch. We then trained the network for 1000 epochs under the same hardware configuration, as previously mentioned in Section 4.4. As the depth estimation and semantic segmentation have a common training procedure and the aim is a dense map, we believe that the two tasks help each other. This is confirmed in Section 5, where the accuracy and error values for the depth are reported; these values were obtained simultaneously with the performance of the semantic segmentation task.

## 5. Results

In this section, we report the noteworthy results obtained with the proposed method. These results indicate the high accuracy of both our semantic segmentation approach and the joint task with depth estimation. We compare our results with those of SOTA methods for semantic segmentation and show that our method outperforms the alternatives by some margin. We also compare the joint task model performance with those of SOTA joint methods.

### 5.1. Semantic Segmentation mIOU Results

**Figure 7:**
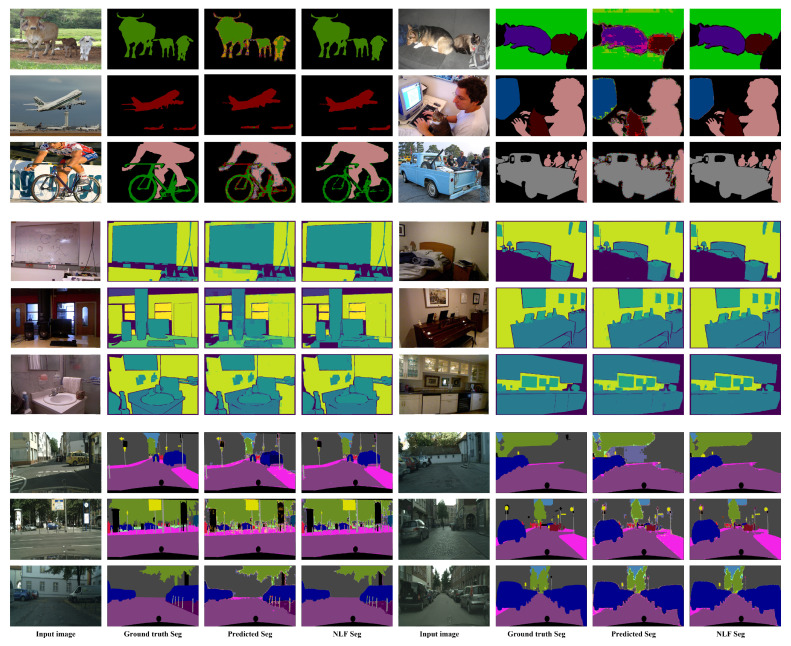
Sample results of predicted and NLF-filtered semantic segmentation (NLF Seg) results that were obtained using DTS-Net on PASCAL VOC2012 (rows 1–3), NYUV2 (rows 4–6), and CITYSCAPES (rows 7–9), with input images and ground-truth segmentation masks (Seg).

Promising mIOU values were exhibited by the proposed method on the three datasets, particularly with the application of the NLF technique. Figure 7 shows sample results from the three datasets with the predicted and filtered (NLF Seg) segmentation results. On the **PASCAL VOC2012** benchmark, DTS-Net and DTS-Net-Lite attained mIOU values of 91.1% and 83.8% on the validation set, respectively. On the **NYUV2** test benchmark, DTS-Net attained high accuracy with a pixel accuracy (Pix.acc.) of 80.72%, while DTS-Net-Lite attained a pixel accuracy (Pix.acc.) of 73.7%. On the **CITYSCAPES** validation benchmark, DTS-Net and DTS-Net-Lite attained mIOU values of 80.72% and 61.5%, respectively. These mIOU and Pix.acc. values were superior to those of all SOTA methods for semantic segmentation, even though the proposed architecture is far simpler than the SOTA-method architectures.

**Figure 8:**
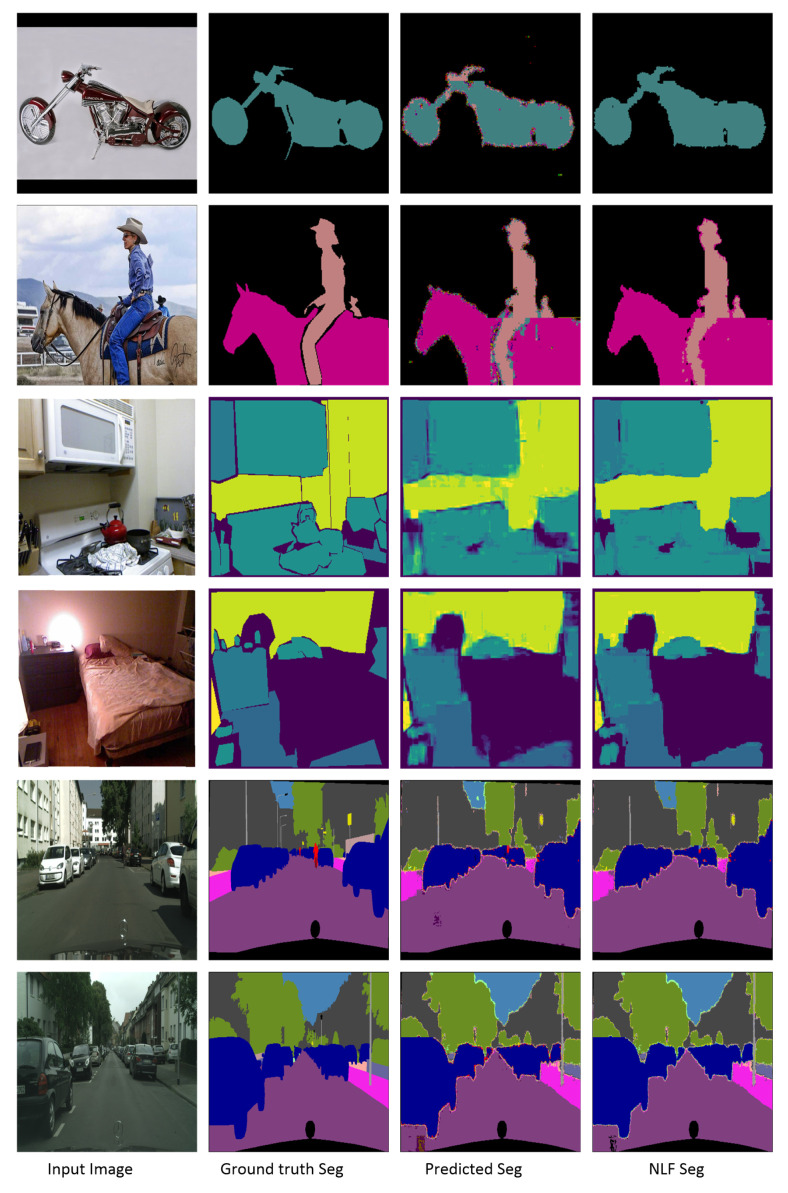
Sample results of predicted and NLF-filtered semantic segmentation (NLF Seg) results that were obtained using DTS-Net-Lite on PASCAL VOC2012 (rows 1 and 2), NYUV2 (rows 3 and 4), and CITYSCAPES (rows 5 and 6) with input images and ground-truth segmentation masks (Seg).

The results in Figure 7 and Figure 8 show the quality of the segmentation results, with fine and sharp edges. Table 2 lists the mIOU values for both DTS-Net and DTS-Net-Lite on the three benchmarks; these results highlight the unique performance of the proposed method. Note that the predicted segmentation masks contained many mispredicted pixel values that had neighboring labels to the true label. Most of those pixels could be corrected easily by the NLF technique. The main reason for the high mIOU and Pix.acc. values obtained by our method is the NLF algorithm which improves the mIOU by 63.4% on PASCAL VOC2012, improves the mIOU by 28.8% on NYUV2, and improves the mIOU by 33.0% on CITYSCAPES. The pixel accuracy (Pix acc.) values also improved when applying NLF but not with a big margin due to the nature of the pixel accuracy metric that reports the number of correctly classified pixels regardless of the ratio of the overlap between the predicted and the ground truth masks which is used in the mIOU metric. Those high improvements in the mIOU and Pix acc. prove the important role of the NLF algorithm in getting more accurate segmentation results than those without NLF.

### 5.2. Semantic Segmentation Speed Results

We measured the processing speed of each frame for both models. Recall that we chose MobileNetV2 as the feature extractor for DTS-Net-Lite to achieve real-time processing. In general, depth-wise separable convolution-based architecture has considerably higher processing speed than normal convolutional architectures, as discussed in detail in Section 3.1. DTS-Net-Lite is faster than DTS-Net because inverted residual and linear bottle-necks are used; however, DTS-Net can achieve an average processing speed. In the experiments, DTS-Net-Lite attained a speed of 19.2 frames per second (fps) with NLF filtration on PASCAL VOC2012 as the image size was 256×256. Without the NLF, it attained 24 fps, as the patch-by-patch filtration required more computations, which consumed more time. DTS-Net with NLF achieved only 17.5 fps on PASCAL VOC2012 and 22 fps without NLF. DTS-Net-Lite achieved speeds of 14.1 and 21.3 fps with and without NLF, respectively, on the NYUV2 dataset. On the CITYSCAPES dataset, DTS-Net-Lite achieved a low speed of 11.7 fps with NLF, as the image size was 512×256, and 14.7 fps without NLF. Table 2 lists the results achieved with DTS-Net and DTS-Net-Lite, that is, the mIOU, the Pix.acc, and the speed.

### 5.3. Comparison with SOTA Semantic Segmentation Methods

**Figure 9:**
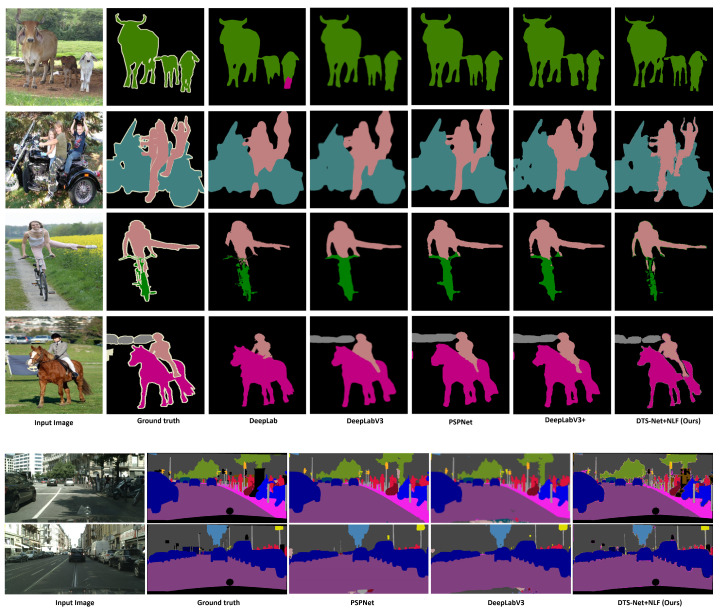
Quality comparison between the semantic segmentation results obtained from the proposed method (DTS-Net+NLF), DeepLab, DeepLabv3, PSPNet, and DeepLabv3+ on PASCAL VOC2012 validation benchmark, and between DTS-Net+NLF, PSPNet, and DeepLabv3 on CITYSCAPES validation benchmarks.

We compared the proposed method with SOTA methods in terms of semantic segmentation for the three benchmarks. On PASCAL VOC2012, the proposed method outperformed the recent SOTA methods in terms of mIOU. In detail, the best-performing SOTA method, that is, EfficientNet-L2+NAS-FPN [21] (based on EfficientNetB7, neural architecture search, and a features pyramid network [38]) achieved an mIOU of 90.0%, whereas DTS-Net achieved 91.1%. Table 3 compares the proposed method and the SOTA methods on the PASCAL VOC2012 validation set in terms of mIOU, and indicates the backbone architecture of each method. Further, Table 4 compares the proposed method with the SOTA methods with regard to semantic segmentation on the NYUV2 benchmark in terms of Pix.acc. Our method DTS-NEt+NLF outperformed the best SOTA method on NYUV2 13 classes (BPNET [39]) by 11.2%, and DTS-NET-Lite+NLF achieves approximately the same performance as BPNET with 0.2% lower pixel accuracy. We also compared our method with the SOTA methods for semantic segmentation on the CITYSCAPES validation benchmark. As detailed in Table 5, the proposed method outperformed the best SOTA method in terms of mIOU by 0.15%. These results prove that the proposed method as a learning technique exhibits higher performance than the recent SOTA encoder-decoder architectures i.e., Deeplabv3, DeepLabv3+ which also depends on depthwise separable convolution architectures (Xception and MobileNet encoders). We also compared the speed of each model (the speeds are copied from the original papers) in Table 5 while the speed comparison is not so informative since the hardware used for testing and the input image size highly affect the speed of the model, and each method uses different hardware specifications and image size. In general, the proposed method exhibited distinctive performance primarily because of the DTS layer which constructs the initial segmentation result and, secondarily, the NLF filtration which enhances this segmentation result. Figure 9 shows a quality comparison between the semantic segmentation results obtained from the best architecture of the proposed method (DTS-Net+NLF) and those of the SOTA methods which have encoder-decoder architecture and some of them have Xception architecture encoder, that is, DeepLabv3 and DeepLabv3+, on PASCAL VOC2012 and CITYSCAPES benchmarks. In Figure 9, the comparison on PASCAL VOC2012 shows that our method can predict a precise segmentation mask even the unlabeled areas (white pixels in the groundtruth) are labeled as background, noting that the borders of our results have few mislabeled pixels. Those pixels exist due to the use of an image reconstruction method (DTS) to construct the segmentation mask, and the NLF algorithm sometimes fails to correct those pixels as their label is a true label of the segmentation output. On a CITYSCAPES quality comparison in Figure 9, our methods can predict high-quality masks with few noisy borders due to the same reasons mentioned at the previous sentence. Note that the black regions contain many mislabeled pixels and this situation is acceptable as the black pixels are unlabeled pixels that are not counted in the segmentation evaluation.

### 5.4. DTS-Net for Joint Depth Estimation and Semantic Segmentation

We trained DTS-Net on the simultaneous joint task of depth estimation and semantic segmentation, as discussed in Section 4.5. The proposed method yielded promising results even though the network performed both tasks simultaneously. The output semantic segmentation and depth estimation had fine details. DTS-Net extracted the important features for each task while using the same feature extractor but with different branches of 1×1×1024 convolutional layers, which were followed by the DTS layer. NLF filtration was then applied to the segmentation output. The network showed promising error values and accuracy for the depth estimation task while attaining a slightly inferior semantic segmentation performance. To evaluate the depth estimation, we evaluated the following depth estimation metrics:

- the absolute relative error (REL): $\frac{1}{n}\sum_{i}^{n}\frac{|y-\hat{y}|}{y}$;
- the relative difference squared (Sq_REL): $\frac{1}{n}\sum_{i}^{n}\frac{||y-\hat{y}{\left|\right|}^{2}}{y}$;
- the root mean squared error (RMSE): $\sqrt{\frac{1}{n}\sum_{i}^{n}{\left(y-\hat{y}\right)}^{2}}$;
- the disparity error (px): $\frac{1}{n}\sum_{i}^{n}\left|y-\hat{y}\right|$;
- the threshold accuracy $\delta_{i}$ of $y_{p}$: $max(\frac{y}{\hat{y}},\frac{\hat{y}}{y})=\delta <thr$ for the commonly used threshold values $thr=1.25,1.25^{2},1.25^{3}$,

where *n* is the number of pixels in the depth map, *i* indicates an iterator over the pixels, *y* and $\hat{y}$ are the ground truth and predicted pixel values, respectively.

**Figure 10:**
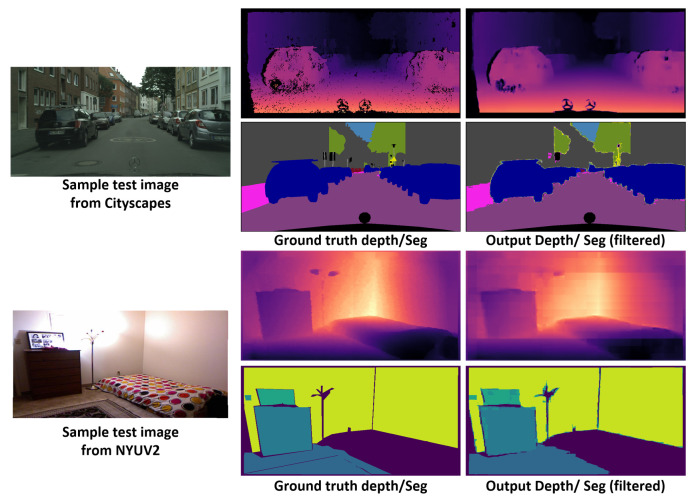
Sample results of the joint semantic segmentation and depth estimation task using DTS-Net. The upper sample is from the CITYSCAPES benchmark and the bottom sample is from the NYUV2 benchmark. To the right of each input image, the ground truth and the output depth and semantic segmentation are given.

The joint trained network yielded high-quality results, as shown in Figure 10. DTS-Net+NLF attained a REL of 0.0987 and $\delta_{1}$ of 90.5% on the CITYSCAPES benchmark. Regarding the semantic segmentation in the joint task, the network attained mIOU values of 72.1% with NLF filtration which is much lower than this for the single task. Further, DTS-Net+NLF attained a REL value of 0.102 and $\delta_{1}$ of 94.59% while attaining an mIOU of 49.1% on the NYUV2 benchmark. The proposed method outperformed the SOTA methods for joint semantic segmentation and depth estimation on the depth estimation by some margin, however, the semantic segmentation was not compared because the segmentation configuration for the models in the comparison is not similar to ours. Table 6 presents the comparison for the depth estimation results with the SOTA methods in terms of REL, Sq Rel, RMSE, the disparity error (px), and the delta accuracies. The proposed method had low depth errors and high delta accuracy for depth estimation surpassing all other joint semantic segmentation and depth estimation accuracies and error values while maintaining a high mIOU for semantic segmentation.

## 6. Future Work

The proposed DTS-Net shows promising results for dense prediction tasks, such as semantic segmentation and depth estimation. In the future, this method can be extended to more challenging dense prediction tasks, such as instance segmentation, through combination with an object detection technique. This approach will allow for discrimination between instances of the same class and can mask those instances with different masks. The feature extractor component could also be replaced with a considerably smaller and simple architecture equivalent to the opposite layer (the space-to-depth), which could then be followed by a small number of convolutional filters. This would reduce the complexity of the feature extractor CNNs. The nearest label filtration (NLF) technique can also be improved using a separate CNN trained on small patches of the training images instead of the integrated branch for classification in current implementation for whole image classification, and the local prediction may attain better filtration accuracy as the current NLF considers the global labels of the image, and not all of those labels exist in every patch of the image.

## 7. Conclusions

The proposed DTS-Net is a simple approach inspired by the DTS image reconstruction technique used in image and video super-resolution. The proposed DTS-Net implementation is simple and yet achieves extremely high accuracy and an average speed for dense prediction tasks such as semantic segmentation, as proven by the results obtained on the different semantic segmentation benchmarks reported herein. For the simultaneous joint task of semantic segmentation and depth estimation, DTS-Net exhibited promising accuracy for both semantic segmentation and depth estimation while the two tasks were trained simultaneously. These results prove the strength of the DTS module, which is the core of the proposed method, and demonstrate its potential for re-employment in dense prediction tasks. The proposed DTS-Net-Lite also provides the ability to perform real-time processing by using a lightweight encoder achieving relatively good segmentation accuracy.

## Figures and Tables

**Table 1:** Comparison of VGG16, ResNet50, DenseNet121, MobileNetV2, and Xception as feature extractors for DTS-Net without filtration in terms of MACs, the number of parameters (Params), and Pix.acc. on the NYUV2 dataset.

Feature Extractor	Params (M)	MACs (G)	Pix.acc. %
VGG16	138.3 M	62.1 G	42.1
DenseNet121	8 M	11.1 G	51.3
ResNet50	25.6 M	15.4 G	62.5
MobileNetV2	**3.5 M**	**1.2 G**	66.7
Xception	22.4 M	18.5 G	**79.4**

**Table 2:** Results that were obtained from DTS-Net and DTS-Net-Lite with and without NLF for each dataset VOC2012, NYUV2, and CITYSCAPES with the mIOU%, Pixel accuracy(Pix.acc.)% and the speed in FPS.

Model	Img Size	Dataset	mIOU%	Pix acc.%	Speed (FPS)
DTS-Net	480 × 480	VOC2012	27.66	90.07	22
DTS-Net	640 × 480	NYUV2	33.92	79.4	18.1
DTS-Net	1024 × 512	CITYSCAPES	47.7	85.1	9.5
DTS-Net-Lite	256 × 256	VOC2012	14.72	87.0	**24**
DTS-Net-Lite	320 × 256	NYUV2	24.5	66.76	21.3
DTS-Net-Lite	512 × 256	CITYSCAPES	35.3	82.8	14.7
DTS-Net+ NLF	480 × 480	VOC2012	**91.1**	**98.8**	15.5
DTS-Net+NLF	640 × 480	NYUV2	62.77	84.73	12.8
DTS-Net+NLF	1024 × 512	CITYSCAPES	80.72	93.1	6.6
DTS-Net-Lite+NLF	256 × 256	VOC2012	83.8	96.1	19.2
DTS-Net-Lite+NLF	320 × 256	NYUV2	48.46	73.3	14.2
DTS-Net-Lite+NLF	512 × 256	CITYSCAPES	61.5	82.06	11.7

**Table 3:** Comparison of the proposed method and SOTA methods on PASCAL VOC2012 validation benchmark.

Method	Backbone	mIOU (%)
DeepLab-CRF [9]	ResNet-101+CRF	77.69
DeepLabv3 [12]	ResNet-101	76.5
DeepLabv3+Res2Net [40]	Res2Net-101	79.3
DeepLabv3+ [13]	Aligned Xception	84.56
PSPNet [15]	RestNet-152	85.4
SpineNet-S143 [24]	Dilated SpineNet	85.64
HPN [19]	ResNet-101	85.8
ExFuse [41]	ResNeXt-131	85.8
Eff-B7 NAS-FPN [42]	EfficientNet-B7+NAS+FPN	86.6
EfficientNet-L2+NAS-FPN [21]	EfficientNet-B7+NAS+FPN	90.0
bf DTS-Net-Lite+NLF	MobileNetV2+DTS	83.8
**DTS-Net+NLF**	Xception+DTS	**91.1**

**Table 4:** Comparison between the proposed method and SOTA methods on the NYUV2 semantic segmentation test benchmark. Note that ScanNet was evaluated on 11 classes only.

Method	Backbone	Pix.acc.%
SceneNet [43]	VGG16	52.5
Hermans et al. [44]	RDF+CRF	54.3
SemanticFusion [45]	VGG16	59.2
ScanNet [46]	NiN [47]	60.7
3DMV [48]	Enet [49]	71.2
BPNet [39]	U-Net	73.5
**DTS-Net-Lite+NLF**	MobileNetV2+DTS	73.3
**DTS-Net+NLF**	Xception+DTS	**84.7**

**Table 5:** Comparison between the proposed method and SOTA methods on CITYSCAPES semantic segmentation validation benchmark in terms of mIOU and speed (FPS).

Method	Backbone	mIOU (%)	Speed (FPS)
DICENet [50]	ShuffleNetV2	63.4	17.2
ContextNet [51]	Shallow+deep CNN	65.9	18.3
Template-Based NAS-arch1 [52]	NAS-Net [53]	69.5	97.0
HPN [19]	ResNet-101	71.7	4.0
FasterSeg [54]	NAS-Net [53]	73.1	6.1
SqueezeNAS [55]	NAS-Net [53]	75.2	0.0065
Dilated-ResNet [11]	Dilated ResNet-101	75.7	-
EEEA-Net-C2 [26]	NAS-Net [53]	76.8	11.6
MDEQ-large [25]	MDEQ	77.8	-
DeepLabv3 [12]	Dilated ResNet-101	78.5	2.0
DeepLabv3+ [13]	Dilated Xception-71	79.6	4.2
PSPNet [15]	Dilated ResNet-101	79.7	1.6
MDEQ-XL [25]	MDEQ	80.3	-
RepVGG-B2 [27]	VGG-like	80.57	4.5
**DTS-Net-Lite+NLF**	MobileNetV2+DTS	61.5	11.7
**DTS-Net+NLF**	Xception+DTS	**80.72**	6.6

**Table 6:** Comparison of the proposed method (DTS-Net+NLF) and the SOTA methods on depth estimation using a joint semantic segmentation and depth estimation model in terms of depth error (REL, Sq Rel, and RMSE), depth accuracy ($\delta_{1}$, $\delta_{2}$, and $\delta_{3}$), and disparity error (px).

Method	Dataset	REL	Sq Rel	RMSE	$\delta_{1}$	$\delta_{2}$	$\delta_{3}$	px
SemDepth+CRF [32]	NYUV2	0.158	0.121	0.641	0.769	0.950	0.988	-
TRL-ResNet50 [33]	NYUV2	0.144	-	0.501	0.815	0.962	0.992	-
HybridNet A2 [34]	CS	0.240	4.27	12.09	0.597	0.822	0.929	-
	NYUV2	0.202	0.186	0.682	0.613	0.892	0.974	-
ESOSD-Net [35]	NYUV2	0.145	-	0.514	0.805	0.962	0.992	-
	CS	-	-	-	-	-	-	2.41
DTS-Net+NLF	NYUV2	0.102	0.503	0.310	0.946	0.997	0.999	-
	CS	0.098	1.093	7.627	0.905	0.956	0.971	1.62

## Data Availability

The datasests used in this paper are public datasets. We also provide the test and the evaluation codes of the proposed method at: https://github.com/HatemHosam/DTS-Net which is created and accessed on 21 June 2021.
